# Multi-target neuroprotection by dl-PHPB in APP/PS1 mice: a proteomic analysis

**DOI:** 10.3389/fphar.2025.1554168

**Published:** 2025-04-24

**Authors:** Yingni Sun, Guoliang Bai, Kangmin Yang, Yong Feng, Hongmei Sun, Li Xian, Hongwei Gao

**Affiliations:** ^1^ School of Life Sciences, Ludong University, Yantai, China; ^2^ Department of Pharmacology, Chinese Academy of Medical Sciences, Beijing, China; ^3^ Beijing Handian Pharmaceutical Co., Ltd., Beijing, China; ^4^ National Center for Pediatric Cancer Surveillance, Beijing Children’s Hospital, Capital Medical University, National Center for Children’s Health, Beijing, China; ^5^ Center for Molecular Cardiology, University of Zürich, Schlieren, Switzerland; ^6^ Department of Medical Research, Qingdao Huangdao District People’s Hospital, Qingdao, China; ^7^ Medicine and Pharmacy Research Center, Binzhou Medical University, Yantai, China

**Keywords:** Alzheimer’s disease, LC-MS/MS, proteomics, dl-PHPB, biomarker

## Abstract

**Introduction:**

Dl-PHPB [potassium 2-(1-hydroxypentyl) benzoate] demonstrates robust neuroprotective effects in preclinical models of Alzheimer’s disease (AD), significantly ameliorating cognitive deficits and pathological hallmarks. However, the underlying mechanism remains largely unclear. The current study primarily focused on elucidating dl-PHPB’s neuroprotective mechanisms and identifying potential targets in preclinical AD models.

**Methods:**

Comparative proteomic analyses were performed on APP/PS1 mice orally administered either dl-PHPB (30 mg/kg) or vehicle daily for 3 months, alongside vehicle-treated wild-type (WT) non-transgenic littermates as controls. Total proteins were separated using two dimensional difference gel electrophoresis, and differentially expressed protein spots were identified via LC‐MS/MS.

**Results and discussion:**

Our results revealed 11 altered proteins in the cortex and 10 in the hippocampus between the WT and APP/PS1 groups treated with vehicle. Following dl-PHPB treatment, 12 differentially expressed proteins were identified in the cortex and 9 in the hippocampus of APP/PS1 mice. These proteins are primarily involved in energy metabolism, neuronal structure, protein trafficking, inflammatory and oxidative responses, and amyloid β (Aβ) and Tau processes, among which several proteins were validated as potential therapeutic targets. Notably, the expression levels of cofilin-2 and VDAC1 in APP/PS1 mice were restored to near-normal levels by the treatment with dl-PHPB, memantine, or donepezil, and further clinical validation is required to establish their utility as AD biomarkers for therapeutic efficacy.

## 1 Introduction

Alzheimer’s disease (AD) is an age-related neurodegenerative disorder characterized by neurofibrillary tangles (NFTs), senile plaques (SPs), and neuronal loss. Memantine and Donepezil are two medications commonly employed in clinical practice for the treatment of AD. Memantine, an NMDA receptor antagonist, protects neurons by blocking glutamate-induced excitotoxicity and is prescribed for moderate to severe cases, improving cognition and behavior. Donepezil, a cholinesterase inhibitor, increases acetylcholine levels, enhancing neurotransmission, and is used for mild to moderate cases, slowing memory and functional decline. Current treatments for AD primarily alleviate clinical symptoms but do not halt or delay the progressive deterioration (Breijyeh and Karaman, 2020; Vaz and Silvestre, 2020; Zhang et al., 2021). There is an urgent need to develop novel therapeutic agents to address this debilitating condition.

Dl-PHPB [potassium 2-(1-hydroxypentyl) benzoate], a novel compound synthesized by the Chinese Academy of Medical Sciences, has shown promising results in preclinical studies. Previous research indicated that dl-PHPB ameliorated neurobehavioral deficits in a cerebral ischemia animal model by enhancing cerebral blood flow and reducing infarct volume (Abidi et al., 2023). In neuroblastoma SK-N-SH cells, dl-PHPB mitigates hydrogen peroxide-induced apoptosis through modulation of the protein kinase C (PKC) signaling pathway (Hu et al., 2012). Additionally, dl-PHPB has been demonstrated to significantly prevent neuropathological changes, inhibit oxidative stress, and reduce neuroinflammatory responses (Zhao et al., 2017; Zhao et al., 2013). Dl-PHPB ameliorated memory deficits and reduced oxidative injury in an AD mouse model by activating Nrf2 signaling pathway (Shang et al., 2024). Recently, it has been reported that dl-PHPB improves learning and memory impairments by inhibiting aberrant tau hyperphosphorylation, restoring impaired long-term potentiation (LTP), and protecting hippocampal neurons, synapses, and dystrophic axons in APP/PS1 transgenic mice (Huang et al., 2019; Li et al., 2014; Peng et al., 2014). These findings suggest that dl-PHPB exerts its neuroprotective effects through multiple mechanisms and may represent a promising therapeutic candidate for the treatment of AD.

Proteomics facilitates discovery-driven research rather than hypothesis testing, thereby enabling the information garnered through proteomics to inform subsequent verification studies. The therapeutic impacts of drugs are manifested through direct or indirect protein modifications. Consequently, proteomics has been utilized to elucidate the molecular mechanisms of multi-target drugs, evaluate their efficacy, and screen for potential drug targets (El-Khateeb et al., 2019; Yokota, 2019; Masuda et al., 2021). The current study aimed to explore the molecular mechanisms underlying the neuroprotective effects of dl-PHPB on cognitive function under pathological conditions using pharmacoproteomics. Additionally, we sought to identify potential anti-AD drug targets of dl-PHPB or candidate biomarkers for AD. To accomplish this, we applied a two-dimensional difference gel electrophoresis approach coupled with mass spectrometry to comprehensively profile differentially expressed proteins in brain tissues affected by dl-PHPB treatment. This unbiased discovery, corroborated by Western blotting validation, facilitated the identification of dl-PHPB-induced protein changes and the exploration of potential drug targets, offering novel insights into the development of anti-AD therapies.

## 2 Materials and methods

### 2.1 Animals and dl-PHPB administration

**FIGURE 1:**
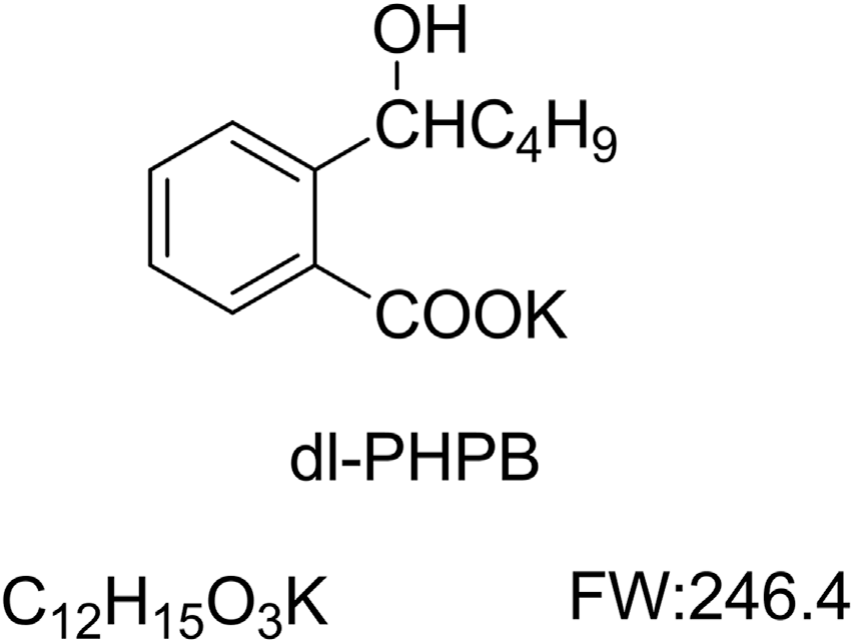
Chemical structure of dl-PHPB.

Dl-PHPB (purity>98%) was synthesized by the Chinese Academy of Medical Sciences (Figure 1). Memantine and donepezil were procured from Sigma-Aldrich and were individually dissolved in distilled water. APP/PS1 double-transgenic mice (strain name: B6C3-Tg (APPswe, PSEN1dE9) 85Dbo/J) were acquired from the Jackson Laboratory. These mice express a chimeric mouse/human amyloid precursor protein (APP) containing the KM670/671NL Swedish mutations and a mutant human presenilin 1 (PS1) carrying the exon 9-deleted variant, both under the control of mouse prion promoter elements, which direct transgene expression primarily to central nervous system neurons (Jankowsky et al., 2001). The two transgenes cosegregate in these mice. The animals were housed in an environmentally controlled room at 23°C ± 1°C with a 12-h light/dark cycle and had free access to water and food. Mice were stratified by weight and randomized into treatment/control groups using a computer-generated sequence. Treatments were administered by blinded personnel via coded syringes. Behavioral tests were scored by an independent observer unaware of group assignments. Sample size was determined using power analysis (α = 0.05, power = 80%, effect size = 1.5 from prior data, and an additional 2–4 rats were included per group to account for attrition. Through the aforementioned design, potential biases are rigorously controlled, thereby strengthening the credibility and reproducibility of the findings while aligning with ARRIVE guidelines for animal research reporting. All experimental procedures were conducted in accordance with the guidelines of the Experimental Animal Center of Ludong University. APP/PS1 transgenic (Tg) mice and age-matched wild-type (WT) mice were randomly assigned to five groups of 12–16 mice each: untreated WT control (WT + H_2_O; n = 16), untreated APP/PS1 Tg model (Tg + H_2_O; n = 14), dl-PHPB-treated Tg group (Tg + dl-PHPB; n = 15), memantine-treated Tg group (Tg + memantine; n = 12), and donepezil-treated Tg group (Tg + donepezil; n = 14). The untreated WT control and Tg model groups received distilled water only. Oral gavage administration began when the mice were 9 months old and continued for 12 weeks. Body weight was monitored biweekly.

### 2.2 Preparation of brain homogenate

The mice were euthanized by CO_2_ inhalation following the completion of behavioral testing. The skin was incised along the midline of the cranium to expose the skull. Curved scissors were used to cut along the cranial sutures from the foramen magnum to the nasal direction, followed by careful removal of the skull. The frontal cortex and hippocampus were microdissected according to the mouse brain stereotaxic atlas and standardized protocols from Nature Protocols 2016. These cortical and hippocampal tissues were immediately snap-frozen in liquid nitrogen and stored at −80°C until further analysis. The tissues were homogenized and processed for protein extraction. Each sample was suspended in 2DE-specific lysis buffer [7 M urea, 2 M thiourea, 4% CHAPS, 1% dithiothreitol (DTT), 0.5% IPG buffer (pH 3–10 NL, GE Healthcare), 1% Protease Inhibitor Mix (Roche), 30 mM Tris-HCl, pH 8.5] and subjected to sonication for 1 min with cycles of 3 s on and 3 s off using a Fisher 550 Sonic Dismembrator. The samples were then centrifuged at 20,000 g at 4°C for 60 min to remove cellular debris. The supernatants were ultrafiltered at 15,000 g for 30 min to eliminate salts and other impurities and were resuspended in 2DE-specific lysis buffer. The resulting protein solutions were collected and stored at −80°C until use. Protein concentrations were quantified using a 2-D Quant Kit (GE Healthcare, United States) according to the manufacturer’s instructions, and separate proteomic analyses were conducted for the cortex and hippocampus.

### 2.3 Step-down passive avoidance test

The impact of dl-PHPB on memory impairment in mice was investigated using a step-down passive avoidance test as previously described (Li et al., 2014; Peng et al., 2014). To minimize locomotor-related confounders, standardized speed measurements were performed on all mice before grouping, and animals with notable locomotor variability were excluded. The apparatus comprised a wooden box measuring 15 × 15 × 30 cm. The floor of the box consisted of parallel stainless steel bars, with a wooden insulating platform (4 × 4 × 4 cm) positioned at the center of the grid floor. When a mouse was placed on the grid floor, it received an inescapable intermittent electric shock (1 s, 36 V), while the platform served as a refuge. The experiment spanned 3 days. On the first day, the mouse was acclimated to the box for 5 min without any electric shock. On the second day, acquisition trials were conducted, during which an electric foot shock (36 V) was administered to the animal’s paws through the grid floor. Upon receiving the shock, the mouse sought refuge on the platform. The training session lasted 3 min. Twenty-four hours after the acquisition trial, the retention test was performed. The error times (frequency of the mouse stepping down from the platform and receiving a shock) and the escape latency (time taken by the mouse to step down from the platform onto the grid floor) were recorded as indicators of retention. If the mouse did not step down to the grid floor within 3 min, a ceiling score of 180 s was assigned. DigBehv-SD (Shanghai Jiliang Software Technology) was used to record and analyze them in the avoidance test.

### 2.4 2DE analysis

Protein samples were loaded onto nonlinear IPG strips (18 cm, pH 3–10 NL, GE Healthcare). Isoelectric focusing was conducted with the following parameters: 50 V for 12 h linearly, 200 V for 1 h linearly, 500 V for 1 h linearly, 1,000 V for 1 h linearly, 8,000 V for 1 h rapidly, and finally reaching a total of 60,000 Vh at 8,000 V. For SDS‒PAGE, the strips were placed on top of a 12.5% SDS‒polyacrylamide gel, and electrophoresis was conducted with the following parameters: 2 W/gel constant power for 1 h, followed by 15 W/gel constant power until the bromophenol blue front reached the bottom of the gel.

### 2.5 Gel staining and image analysis

After performing 2-DE, the analytical gels were silver-stained using the PlusOne Silver Staining Kit (GE Healthcare). The silver-stained gels were then scanned with an ImageScanner III (GE Healthcare). Image analysis was conducted using ImageMaster 2D Platinum software version 7.0 (GE Healthcare). Protein spots were identified based on the following criteria: a two-fold greater difference in protein expression between the two groups (ratio>2). Statistical significance was assessed using the Student’s t-test with a threshold of FDR-adjusted *p* < 0.05. The symbols “+” and “−” denote upregulation and downregulation, respectively.

### 2.6 Protein identification by nano-HPLC-ESI-MS/MS

The differentially expressed protein spots were manually excised, reduced, alkylated, and subsequently digested using sequence-grade modified trypsin (Promega Corporation, United States). LC‒MS/MS analysis was conducted using a Surveyor MS Plus HPLC system coupled to a ThermoFinnigan LTQ linear ion trap mass spectrometer (ThermoFisher Corporation, San Jose, CA). The resulting tryptic peptides were loaded onto a trap column (300SB-C18, 5 × 0.3 mm, 5 μm particle size) (Agilent Technologies, Santa Clara, CA) connected via a zero-dead-volume union to a self-packed analytical column (C18, 100 × 0.1 mm, 3 μm particle size) (SunChrom, Germany). Peptides were eluted with a linear acetonitrile gradient at a flow rate of 500 nL/min. Data-dependent scanning was employed to select the five most abundant ions from a full-scan mass spectrum for fragmentation by collision-induced dissociation. MS data were analyzed using SEQUEST against the NCBI Reference Sequence mouse protein database, and the results were filtered, sorted, and displayed using Bioworks 3.2. Protein lists were filtered based on the following criteria: peptide Xcorr values >1.90 (for +1 charge), >2.75 (for +2 charge), >3.75 (for +3 charge), peptide ΔCN >0.1, and protein probabilities <0.001. Each identified protein required at least two unique peptides.

### 2.7 Western blotting

Seven proteins—pyruvate dehydrogenase E1 component subunit α (PDHE1α), voltage-dependent anion-selective channel protein 1 (VDAC1), dihydropyrimidinase-related protein 2 (DRP-2), cofilin-2, peroxiredoxin-6, peptidyl-prolyl cis-trans isomerase NIMA-interacting 1 (Pin 1), and cathepsin-B—were validated by Western blot analysis. Equal amounts of protein (40 μg) were separated on a 10% polyacrylamide gel, transferred onto a polyvinylidene difluoride (PVDF) membrane, and incubated with primary antibodies overnight. The following antibodies were used: mouse anti-β-actin (1:10,000; Sigma), rabbit anti-PDHE1α (1:200; Santa Cruz Biotechnology), rabbit anti-VDAC1 (1:1,000; Abcam), rabbit anti-DRP-2 (1:1,000; Cell Signaling Technology), mouse anti-cofilin-2 (1:200; Santa Cruz Biotechnology), rabbit anti-peroxiredoxin-6 (1:1,000; Abcam), rabbit anti-Pin 1 (1:200; Santa Cruz Biotechnology), and rabbit anti-cathepsin B (1:200; Santa Cruz Biotechnology). The signals were detected using an enhanced chemiluminescence (ECL) system (LAS-3000 Luminescent Image Analyzer, Fujifilm) and quantified using Quantity One software. The values were normalized to the intensity of β-actin.

### 2.8 Statistical analysis

The normalized volume of each spot in proteomics was compared between the two groups using Student’s t-test. To control family-wise error rate, Benjamini–Hochberg false discovery rate (FDR) correction was applied to all pairwise comparisons (q < 0.05). Expression changes were considered biologically significant only when meeting both criteria: (a) FDR-adjusted *p* < 0.05 and (b) fold-change>2. In Western blot analysis and behavioral testing results, one-way analysis of variance (ANOVA) was conducted using SPSS 13.0 software. Data were presented as means±SEMs, and statistical significance was set at *p* < 0.05.

## 3 Results

### 3.1 Dl-PHPB ameliorated memory deficits in APP/PS1 transgenic mice

**FIGURE 2:**
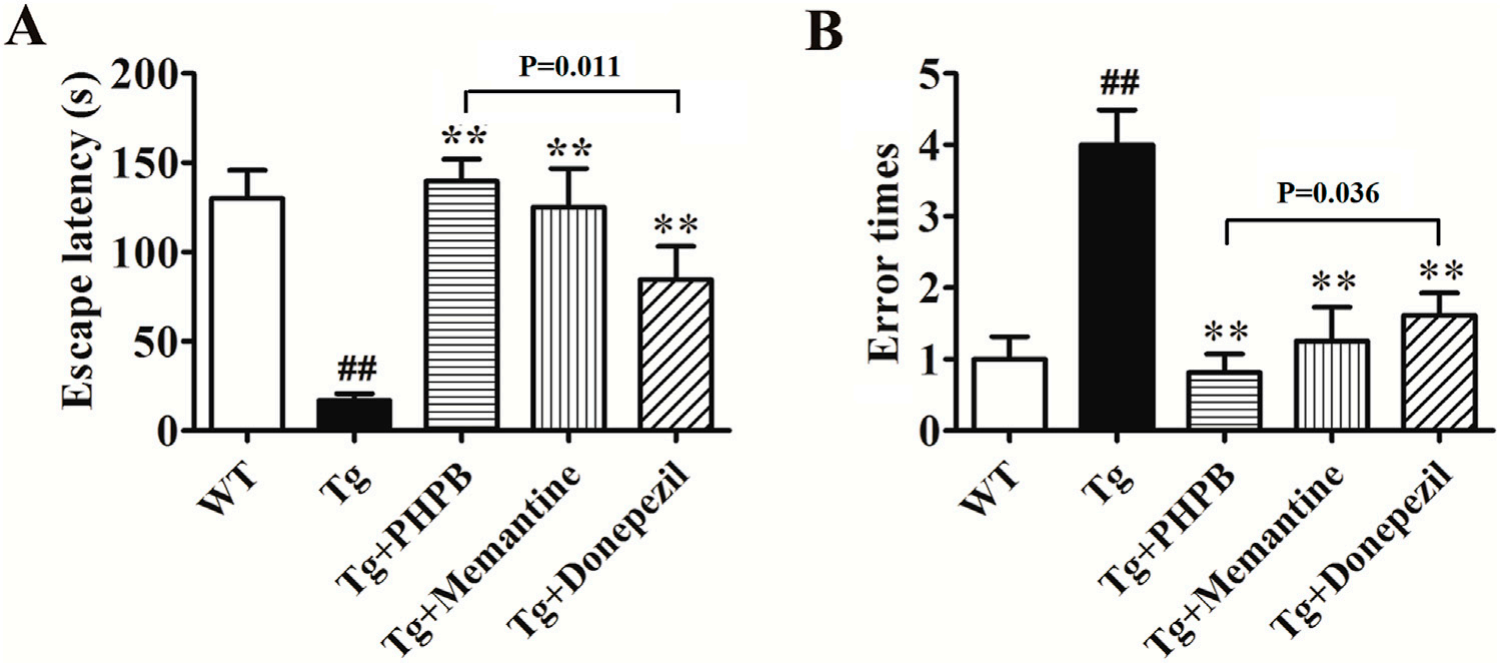
Long-term dl-PHPB treatment ameliorated spatial learning and memory deficits in APP/PS1 mice. The latency **(A)** and error times **(B)** were evaluated using the step-down passive avoidance test. Compared with vehicle treatment, dl-PHPB, memantine, and donepezil treatments significantly attenuated learning and memory deficits in APP/PS1 mice. Statistical analysis: Between-group differences were analyzed by one-way ANOVA followed by Tukey’s post hoc test for multiple comparisons. Data were presented as mean ± SEM (n = 12–16). ^##^ *p* < 0.01 vs. vehicle-treated WT group; **p < 0.01 vs. vehicle-treated APP/PS1 group.

In this study, we assessed whether dl-PHPB could mitigate behavioral deficits in APP/PS1 mice using the step-down passive avoidance test. Following a 3-month oral administration of dl-PHPB, memantine, or donepezil, behavioral tests were conducted. Escape latency and error times were recorded during the retention test session. A significant difference in escape latency and error times (p < 0.01) was observed between vehicle-treated WT and vehicle-treated Tg groups, indicating that APP/PS1 mice exhibited pronounced memory deficits. Specifically, the escape latency results showed that vehicle-treated Tg mice spent significantly less time on the platform compared to vehicle-treated WT mice (Tg: 16.9 ± 3.8 s; WT: 130.1 ± 15.6 s; Figure 2A). Treatment with dl-PHPB, memantine, and donepezil significantly prolonged escape latency (Tg + dl-PHPB: 139.7 ± 12.3 s; Tg + Memantine: 125.3 ± 21.4 s; Tg + Donepezil: 84.5 ± 18.7 s; Figure 2A) in APP/PS1 mice. Additionally, the vehicle-treated Tg group made more errors (Tg: 4.0 ± 0.5; WT: 1.0 ± 0.3; Figure 2B) compared to the vehicle-treated WT group, and long-term oral treatment with dl-PHPB, memantine, and donepezil reduced the error frequency (Tg + dl-PHPB: 0.8 ± 0.3; Tg + Memantine: 1.3 ± 0.5; Tg + Donepezil: 1.6 ± 0.3; Figure 2B) in APP/PS1 mice.

### 3.2 Pharmacoproteomic analysis of dl-PHPB in APP/PS1 mice

**FIGURE 3:**
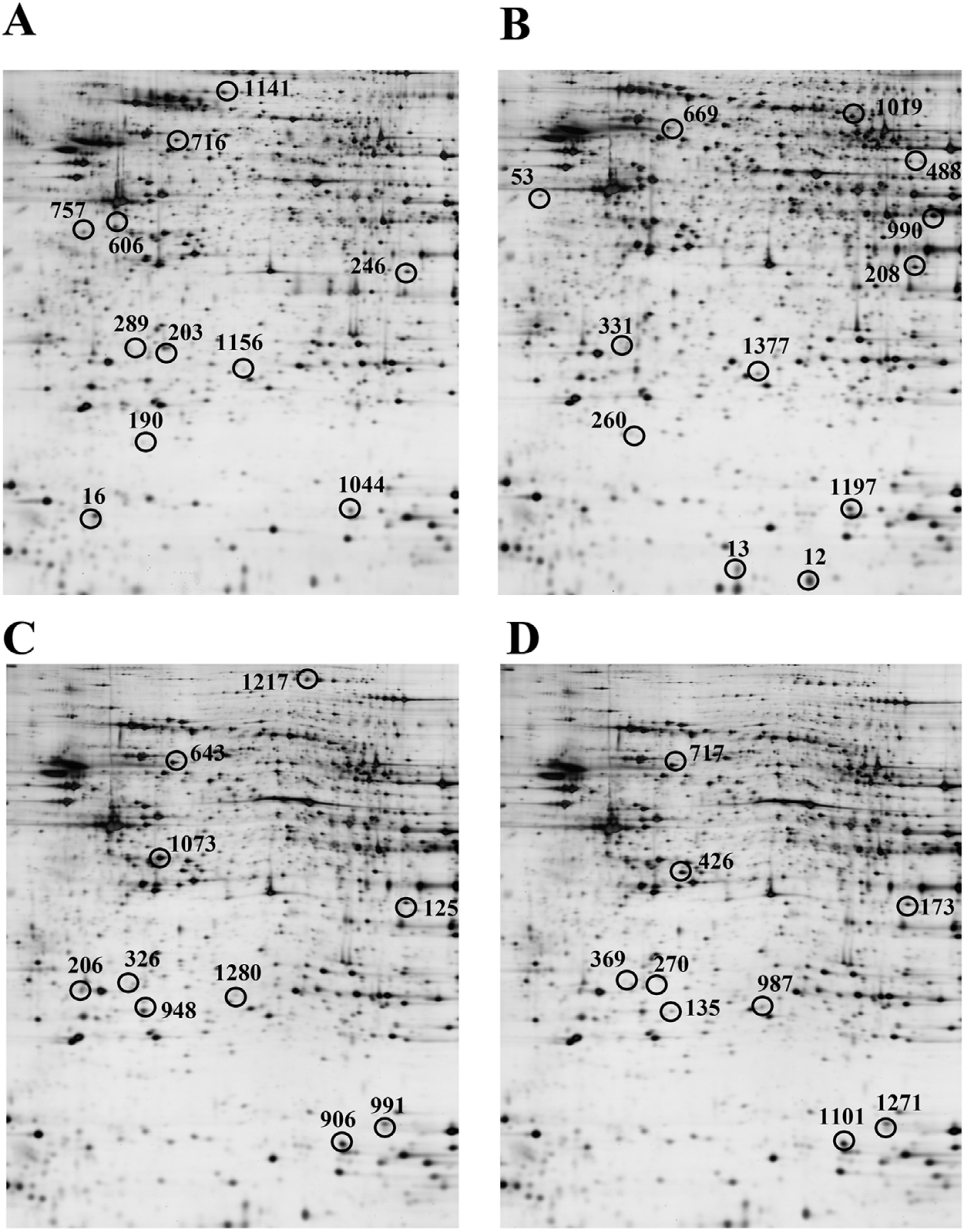
Representative 2D gel images of differentially expressed proteins in the cortex **(A,B)** and hippocampus **(C,D)** across groups. A and C, APP/PS1+H_2_O vs. WT + H_2_O. B and D, APP/PS1+dl-PHPB vs. APP/PS1+H_2_O. Approximately 120 µg of protein was loaded per gel to detect protein expression. n = 10. Differentially expressed protein spots (ratio>2, q < 0.05) were labeled in the images. Detailed identification information was shown in Tables 1–4, respectively.

Proteomic analyses using 2DE, in-gel trypsin digestion, and LC‒MS/MS were conducted on homogenates from the cortex and hippocampus of WT mice provided with drinking water and APP/PS1 mice given either drinking water or water supplemented with dl-PHPB for a period of 3 months. The comparisons included: (i) APP/PS1+H_2_O vs. WT + H_2_O and (ii) APP/PS1+dl-PHPB vs. APP/PS1+H_2_O. Protein spots that exhibited differential expression and were identified through LC‒MS/MS are labeled in Figure 3 and detailed in Tables 1–4. Identified proteins were classified based on their functions, which primarily encompassed the regulation of energy metabolism, neuronal structure and protein trafficking, inflammatory and oxidative responses, and Aβ and Tau processes (Table 5).

**TABLE 1:** Identification data of cortical proteins significantly altered in APP/PS1 vs. WT mice (H_2_O-treated).

Spot No.^a^	Protein identity	Accession GI no.	Sequence Coverage (%)	Score	pI^b^	MW^c^	Variation ratio^d^
16	astrocytic phosphoprotein FEA-15 isoform 2	21426847	36.6	40.2	4.80	15054.0	−2.01
190	peroxiredoxin-6	6671549	56.1	110.3	5.96	24826.4	+2.66
203	malate dehydrogenase	254540027	30.9	130.2	6.16	36510.9	−2.43
246	pyruvate dehydrogenase E1 component subunit α (PDHE1α)	6679261	25.7	80.3	8.49	43198.6	−2.01
289	cathepsin B	309152	28.8	70.2	5.51	37279.6	+3.22
606	glial fibrillary acidic protein isoform 1	196115327	47.7	288.3	5.31	49364.2	+3.34
	glial fibrillary acidic protein isoform 2	84000448	46.5	288.3	5.14	49899.7	
716	dihydropyrimidinase-related protein 2 (DRP-2)	40254595	23.4	60.2	5.93	62278.1	+2.12
757	γ-enolase	7305027	27.6	90.3	4.84	47296.3	−2.27
1044	peptidyl-prolyl cis-trans isomerase NIMA-interacting 1 (Pin 1)	20139259	57.9	180.2	7.97	17971.2	−2.75
1141	ezrin	83921618	12.6	80.2	5.76	69406.3	+2.16
1156	voltage-dependent anion-selective channel protein 1 (VDAC1)	6755963	16.8	90.3	8.86	30755.3	+2.78

^a^ Spot numbers correspond to those shown in Figure 3A.

^b^ pI, isoelectric point.

^c^ MW, molecular mass.

^d^ “+” and “–” represent “upregulation” and “downregulation”, respectively. FDR-adjusted *p* < 0.05.

**TABLE 2:** Identification data of hippocampal proteins significantly altered in APP/PS1 vs. WT mice (H_2_O-treated).

Spot No.^a^	Protein identity	Accession GI no.	Sequence Coverage (%)	Score	pI^b^	MW^c^	Variation ratio^d^
125	pyruvate dehydrogenase E1 component subunit α (PDHE1α)	6,679,261	23.9	60.2	8.49	43198.6	−2.21
206	triosephosphate isomerase	226958349	26.7	100.3	4.74	32192.5	−2.03
326	cathepsin B	309152	40.5	140.2	5.51	37279.6	+3.69
643	dihydropyrimidinase-related protein 2 (DRP-2)	40254595	29.1	70.3	5.93	62278.1	+2.32
906	peptidyl-prolyl cis-trans isomerase NIMA-interacting 1 (Pin 1)	20139259	46.6	120.2	7.97	17971.2	−2.45
948	pyridoxal kinase	26006861	12.9	30.2	5.86	35015.0	−2.56
991	cofilin-2	6671746	18.9	30.2	8.12	18709.5	+2.49
1073	D-lactate dehydrogenase, mitochondrial	23506790	13.5	40.2	6.16	51847.6	−2.06
1217	dynamin-1	116063570	30.3	180.3	7.61	97283.7	−2.77
1280	voltage-dependent anion-selective channel protein 1 (VDAC1)	6755963	61.0	328.3	8.86	30755.3	+3.82

^a^ Spot numbers correspond to those shown in Figure 3C.

^b^ pI, isoelectric point.

^c^ MW, molecular mass.

^d^ “+” and “–” represent “upregulation” and “downregulation”, respectively. FDR-adjusted *p* < 0.05.

**TABLE 3:** Identification data of cortical proteins significantly altered by dl-PHPB vs. H_2_O in APP/PS1 mice.

Spot No.^a^	Protein identity	Accession GI no.	Sequence Coverage (%)	Score	pI^b^	MW^c^	Variation ratio^d^
12	histidine triad nucleotide-binding protein 1	33468857	20.4	20.2	6.41	13776.8	+2.01
13	fatty acid-binding protein	6754450	29.2	38.2	6.15	15137.3	+2.24
53	tubulin	34740335	6.65	20.2	4.81	50151.3	−2.17
208	pyruvate dehydrogenase E1 component subunit α (PDHE1α)	6679261	28.1	100.3	8.49	43198.6	+2.68
260	peroxiredoxin-6	6671549	40.7	90.3	5.96	24826.4	+2.33
331	cathepsin B	309152	14.3	30.2	5.51	37279.6	−2.10
488	isocitrate dehydrogenase	1236984	15.5	40.2	8.89	58749.1	+2.02
669	dihydropyrimidinase-related protein 2 (DRP-2)	40254595	38.6	150.2	5.93	62278.1	−2.24
990	dihydrolipoamide succinyltransferase, mitochondrial	21313536	26.7	80.2	9.10	48994.5	+2.66
1019	dihydropyrimidinase-related protein 5	12746424	22.8	80.2	6.65	61516.1	+2.12
1197	peptidyl-prolyl cis-trans isomerase NIMA-interacting 1 (Pin 1)	20139259	38.4	108.3	7.97	17971.2	+3.01
1377	voltage-dependent anion-selective channel protein 1 (VDAC1)	6755963	36.5	180.3	8.86	30755.3	−3.08

^a^ Spot numbers correspond to those shown in Figure 3B.

^b^ pI, isoelectric point.

^c^ MW, molecular mass.

^d^ “+” and “–” represent “upregulation” and “downregulation”, respectively. FDR-adjusted *p* < 0.05.

**TABLE 4:** Identification data of hippocampal proteins significantly altered by dl-PHPB vs. H_2_O in APP/PS1 mice.

Spot No.^a^	Protein identity	Accession GI no.	Sequence Coverage (%)	Score	pI^b^	MW^c^	Variation ratio^d^
135	carbonic anhydrase 2	157951596	34.9	50.3	6.54	29032.3	−2.25
173	pyruvate dehydrogenase E1 component subunit α (PDHE1α)	6679261	20.4	50.3	8.49	43198.6	+2.41
270	malate dehydrogenase	254540027	21.7	90.2	6.16	36510.9	+2.07
369	cathepsin B	309152	34.3	160.2	5.51	37279.6	−3.13
426	α-enolase	158853992	27.6	88.3	6.70	47618.1	+2.54
717	dihydropyrimidinase-related protein 2 (DRP-2)	40254595	33.5	110.2	5.93	62278.1	−2.21
987	voltage-dependent anion-selective channel protein 1 (VDAC1)	6755963	50.2	252.4	8.86	30755.3	−2.52
1101	peptidyl-prolyl cis-trans isomerase NIMA-interacting 1 (Pin 1)	20139259	54.3	150.2	7.97	17971.2	+2.19
1271	cofilin-2	6671746	18.9	20.2	8.12	18709.5	−2.96

^a^ Spot numbers correspond to those shown in Figure 3D.

^b^ pI, isoelectric point.

^c^ MW, molecular mass.

^d^ “+” and “–” represent “upregulation” and “downregulation”, respectively. FDR-adjusted *p* < 0.05.

**TABLE 5:** Functional roles attributed to identified proteins.

Function	Identified proteins
Energy metabolism	VDAC1; PDHE1α; D-lactate dehychgenase, mitochondrial; malate dehydrogenase; α-enolase; γ-enolase; triosephosphate isomerase; isocitrate dehydrogenase; dihydrolipoamide succinyltransferase, mitochondrial
Neuronal structure and protein trafficking	DRP-2; cofilin-2; tubulin; dihydropyrimidinase-related protein 5; dynamin-1; histidine triad nucleotide-binding protein 1; carbonic anhydrase 2; fatty acid-binding protein
Inflammatory and oxidative response	peroxiredoxin-6; stress-70 protein; ezrin, astrocytic phosphoprotein FEA-15 isoform 2; glial fibrillary acidic protein
Aβ and Tau process	Pin 1; cathepsin B
Others	pyridoxal kinase

We identified 11 differentially expressed proteins in the cortex and 10 in the hippocampus between vehicle-treated WT and vehicle-treated APP/PS1 mice (Tables 1, 2). In the cortex, 6 proteins (peroxiredoxin-6, cathepsin B, glial fibrillary acidic protein, DRP-2, ezrin, and VDAC1 showed increased expression, while 5 proteins (astrocytic phosphoprotein FEA-15 isoform 2, malate dehydrogenase, PDHE1α, γ-enolase, and Pin 1) exhibited decreased expression. In the hippocampus of APP/PS1 mice, compared to WT mice, 4 proteins (cathepsin B, DRP-2, cofilin-2, and VDAC1) were upregulated, whereas 6 proteins (PDHE1α, triosephosphate isomerase, Pin 1, pyridoxal kinase, D-lactate dehydrogenase, and mitochondrial dynamin-1) were downregulated.

Proteomic analyses revealed differential expression of 12 cortical and 9 hippocampal proteins between dl-PHPB- and vehicle-treated APP/PS1 mice. In the cortex, the expression levels of histidine triad nucleotide-binding protein 1, fatty acid-binding protein, PDHE1α, peroxiredoxin-6, isocitrate dehydrogenase, dihydrolipoamide succinyltransferase, dihydropyrimidinase-related protein 5, and Pin 1 were significantly elevated, while those of tubulin, cathepsin B, DRP-2, and VDAC1 were significantly reduced in dl-PHPB-treated APP/PS1 Tg mice compared to vehicle-treated APP/PS1 Tg mice. In the hippocampus, four proteins (PDHE1α, malate dehydrogenase, α-enolase, and Pin 1) showed significant increases, whereas five proteins (carbonic anhydrase 2, cathepsin B, DRP-2, VDAC1, and cofilin-2) exhibited decreased levels following dl-PHPB treatment.

### 3.3 Western blotting analysis

**FIGURE 4:**
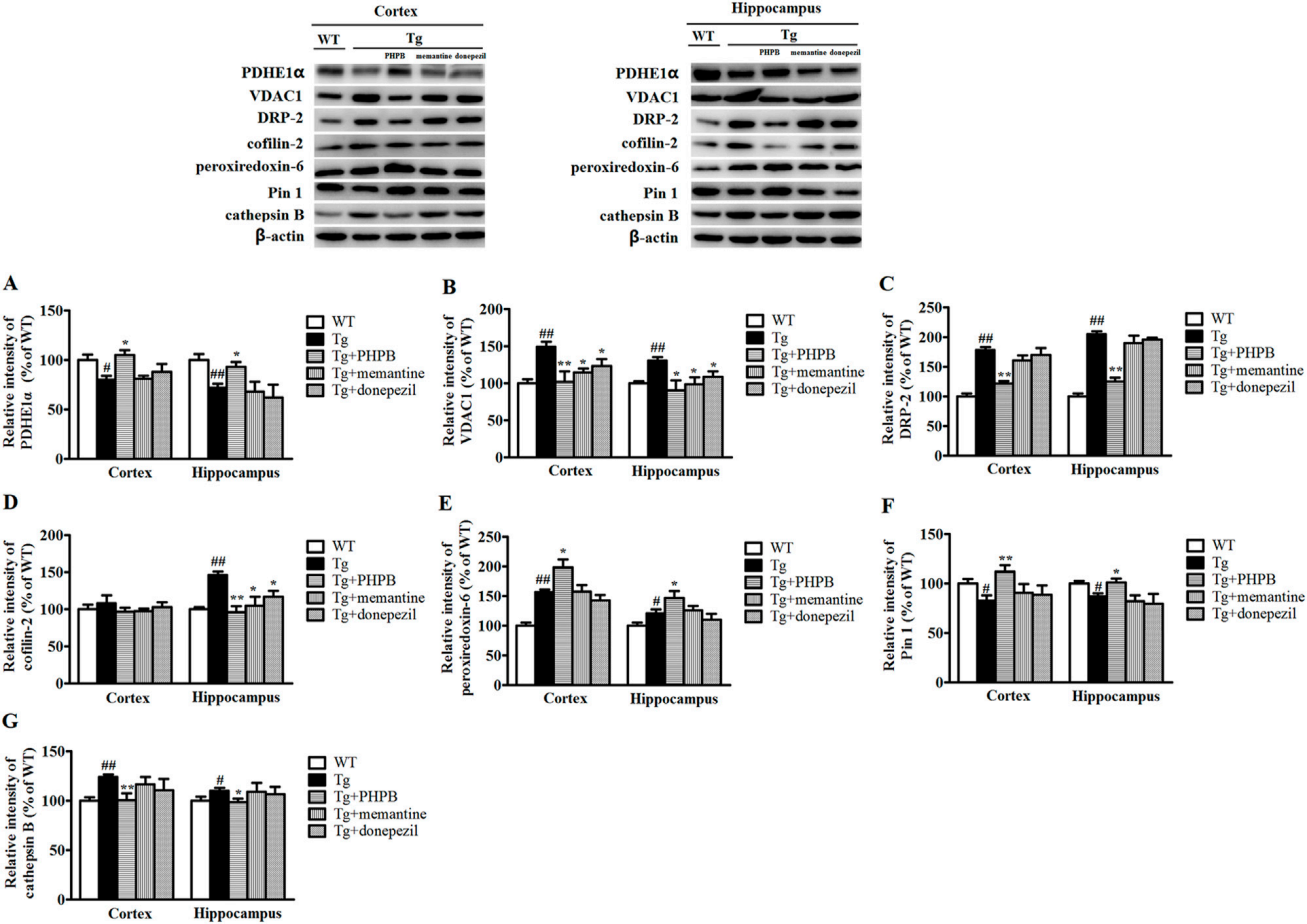
Western blot analysis of several proteins with altered abundance in the cortex or hippocampus of WT + H_2_O, APP/PS1+H_2_O, APP/PS1+dl-PHPB, APP/PS1+memantine, and APP/PS1+donepezil mice. The changes in PDHE1α **(A)**, VDAC1 **(B)**, DRP-2 **(C)**, cofilin-2 **(D)**, peroxiredoxin-6 **(E)**, Pin 1 **(F)**, and cathepsin B **(G)** detected by Western blotting were consistent with the proteomic data. The Western blot images correspond to: lane 1, WT mice treated with normal drinking water; lane 2, APP/PS1 mice treated with normal drinking water; lane 3, APP/PS1 mice treated with dl-PHPB; lane 4, APP/PS1 mice treated with memantine; and lane 5, APP/PS1 mice treated with donepezil. Quantified results were normalized to β-actin expression. Values were expressed as percentages relative to vehicle-treated WT mice (WT + H_2_O) (set to 100%) and presented as the group mean ± SEM. Statistical analysis: Between-group differences were analyzed by one-way ANOVA followed by Tukey’s post hoc test for multiple comparisons. n = 8–10 mice per group. ^#^ *p* < 0.05, ^##^ *p* < 0.01 versus the vehicle-treated WT group. *p < 0.05, **p < 0.01 versus the vehicle-treated APP/PS1 group.

Western blotting analysis was conducted to validate alterations in the protein expression of PDHE1α, VDAC1, DRP-2, cofilin-2, peroxiredoxin-6, Pin 1, and cathepsin B in APP/PS1 mice following long-term dl-PHPB treatment. As illustrated in Figure 4, the levels of VDAC1, DRP-2, and cathepsin B were significantly elevated in the cortex and hippocampus of vehicle-treated APP/PS1 mice compared to WT controls. Conversely, dl-PHPB treatment markedly reduced the expression of these proteins in APP/PS1 mice relative to vehicle-treated APP/PS1 mice (Figures 4B,C,G). Similarly, dl-PHPB treatment significantly decreased hippocampal cofilin-2 levels but had no significant effect on cortical cofilin-2 levels in APP/PS1 mice (Figure 4D). Compared to WT mice, a substantial decrease in PDHE1α and Pin 1 expression was observed in the cortex and hippocampus of APP/PS1 mice, and this reduction was significantly reversed by dl-PHPB treatment (Figures 4A,F). Notably, the expression levels of peroxiredoxin-6 were significantly higher in the cortex and hippocampus of APP/PS1 mice compared to WT controls, and dl-PHPB treatment further increased this expression (Figure 4E). The Western blot results were generally consistent with the proteomic analysis, thereby validating the reliability of the proteomics method.

To examine the impact of positive AD drugs on differentially expressed proteins, mice in the treatment groups were orally administered memantine and donepezil, respectively. Quantitative analysis of Western blot bands revealed a significant increase in VDAC1 levels in both the cortex and hippocampus, as well as a notable rise in cofilin-2 levels specifically in the hippocampus. These increases were markedly attenuated by memantine or donepezil treatments (Figures 4B,D). Furthermore, Western blot results showed no statistically significant alterations in the protein levels of PDHE1α, DRP-2, peroxiredoxin-6, Pin-1, and cathepsin B following either memantine or donepezil treatment, which may be attributed to their single-target mechanisms (Figures 4A,C,E–G).

## 4 Discussion

The hallmark of AD is cognitive decline associated with Aβ deposition. Extensive research has demonstrated that APP/PS1 transgenic mice exhibit robust amyloid plaque pathology akin to that observed in AD and develop age-related memory deficits (Trinchese et al., 2004). The step-down passive avoidance task, a fear-motivated test, is commonly employed to assess learning and memory in rodent models of central nervous system disorders. Notably, this test revealed that oral administration of dl-PHPB for 3 months significantly ameliorated memory impairment in APP/PS1 mice. These protective effects on learning and memory are consistent with our previous findings (Li et al., 2014; Peng et al., 2014).

In the present study, we conducted a comprehensive proteomic analysis of the cortex and hippocampus in vehicle-treated WT, vehicle-treated, and dl-PHPB-treated APP/PS1 mice. Analysis of over 1,300 spots on 2DE gels identified 11 differentially expressed spots in the cortex and 10 in the hippocampus between the WT + H_2_O and APP/PS1+H_2_O groups; 12 spots were different in the cortex and 9 in the hippocampus between the APP/PS1+dl-PHPB and APP/PS1+H_2_O groups. Western blot analysis confirmed the proteomic findings. The identified proteins play roles in energy metabolism, neuronal structure, protein trafficking, inflammatory and oxidative responses, as well as Aβ and tau processes.

Disturbances in energy metabolism have been well-documented in AD brains (Hoyer, 2004). This is substantiated by a concurrent reduction in glucose utilization and the expression of glycolytic enzymes (Hoyer et al., 1991). In our present investigation, we observed a marked downregulation of key metabolic proteins, including PDHE1α, malate dehydrogenase, D-lactate dehydrogenase, γ-enolase, and triosephosphate isomerase, in APP/PS1 mice relative to WT mice. These reductions imply a potential impairment in energy metabolism, which could compromise the cognitive function of the brain, given its substantial reliance on glucose for ATP production. PDHE1α is an essential component of pyruvate dehydrogenase, governing its activity (Korotchkina et al., 1995). As the rate-limiting enzyme in oxygen-dependent energy production, pyruvate dehydrogenase catalyzes the conversion of pyruvate to acetyl coenzyme A, bridging cytosolic glycolysis to the mitochondrial citric acid cycle (Pithukpakorn, 2005). Consequently, PDHE1α plays a pivotal role in mitochondrial energetics. Research has indicated a decline in PDHE1α protein levels and activity in the early stages of AD model mice and in rats with traumatic brain injury (Sharma et al., 2009; Yao et al., 2009; Xing et al., 2009). In AD neurons, the deficiency of PDHE1α results in diminished glucose aerobic oxidation, leading to reduced oxidative phosphorylation and energy failure (Stacpoole, 2012). Furthermore, the reduction in PDHE1α diminishes the production of acetyl coenzyme A, thereby decreasing the synthesis of the neurotransmitter acetylcholine (Ronowska et al., 2018). Our findings suggest that treatment with dl-PHPB significantly upregulates PDHE1α and other glucose-metabolizing enzymes (α-enolase, malate dehydrogenase, isocitrate dehydrogenase, and dihydrolipoyl succinyltransferase), indicating that dl-PHPB may enhance energy metabolism in APP/PS1 mouse brains, ultimately ameliorating cognitive function.

In addition, our findings indicate that the expression of VDAC1 was markedly elevated in APP/PS1 mice compared to WT mice, consistent with our prior research (Sun et al., 2014). Previous studies have demonstrated that VDAC1 expression is significantly upregulated in the cerebral cortices of 6-, 12-, and 24-month-old APP transgenic mice relative to age-matched WT controls (Manczak and Reddy, 2012). Furthermore, elevated VDAC1 levels have been documented in the affected brain regions of AD postmortem samples (Yoo et al., 2001; Manczak and Reddy, 2012) and in the serum of AD patients (Sun et al., 2014). VDAC1 is recognized as a key regulator of mitochondrial function (Lemasters and Holmuhamedov, 2006; Choudhary et al., 2014; Shoshan-Barmatz et al., 2018), and plays a critical role in various cellular processes, particularly mitochondrial energy metabolism. It is hypothesized that elevated VDAC1 directly interacts with Aβ and phosphorylated tau in neurons, leading to the obstruction of mitochondrial pores and disruption of metabolite transport, which ultimately results in mitochondrial dysfunction and impaired ATP supply to nerve terminals (Manczak and Reddy, 2012). In the present study, we observed that treatment with dl-PHPB, memantine, or donepezil significantly reduced VDAC1 levels in the cortex and hippocampus of APP/PS1 mice compared to vehicle-treated APP/PS1 mice. By decreasing VDAC1 levels, normal mitochondrial pore dynamics can be restored, thereby enhancing energy production.

Neuronal and synaptic degeneration are key pathological features of AD; therefore, proteins involved in neuronal structure and trafficking likely play crucial roles in AD pathogenesis. DRP-2, also known as collapsin response mediator protein 2 (CRMP-2), is implicated in neurite growth, guidance, and neuronal development and polarity (O'Donnell et al., 2009; Ip et al., 2014). Our findings demonstrated that DRP-2 expression was significantly elevated in the cortex and hippocampus of vehicle-treated APP/PS1 mice compared to vehicle-treated WT mice. DRP-2 has been observed to colocalize with neurofibrillary tangles in the cortex of AD patients (Yoshida et al., 1998) and can be phosphorylated by cyclin-dependent kinase 5 (CDK5), glycogen synthase kinase 3β (GSK3β), Rho kinase, and calmodulin-dependent protein kinase II (CaMKII), which inhibit axonal growth and vesicle trafficking (Gu et al., 2000; Yoshimura et al., 2005; Schmidt and Strittmatter, 2007; Hou et al., 2009). Additionally, increased phosphorylation of DRP-2 has been reported to precede AD pathology (Cole et al., 2007). In our study, the overexpression of DRP-2 may be associated with neuritic reorganization and the formation of dystrophic neurites surrounding amyloid plaques. Treatment with dl-PHPB significantly reduced the elevated DRP-2 expression in APP/PS1 mice, potentially contributing to the maintenance of neurite cytoskeletal integrity.

Cofilin, the principal actin-depolymerizing factor, plays a critical role in learning and memory (Kang and Woo, 2019). Prior researches have demonstrated that cofilin and actin can form rod-like structures around amyloid plaques, disrupting distal neurite function and ultimately leading to neuritic atrophy and cell death (Minamide et al., 2000; Bamburg and Bernstein, 2016). Moreover, cofilin regulates α-amino-3-hydroxy-5-methyl-4-isoxazolepropionic acid (AMPA) and N-methyl-D-aspartate (NMDA) receptor-dependent dendritic spine plasticity (Pontrello et al., 2012; Gu et al., 2010), thereby influencing learning and memory. Recent studies have indicated that cofilin activation exacerbates tau pathology by impairing tau-mediated microtubule dynamics, thus affecting learning and memory (Woo et al., 2019). In AD, cofilin has been reported to mediate neuronal apoptosis through p53 translocation and phospholipase D1 (PLD1) regulation (Liu et al., 2017). Our previous findings have shown a substantial increase in cofilin-2 levels in the hippocampus of APP/PS1 mice (Sun et al., 2014). In the present study, long-term treatment with dl-PHPB markedly prevented this increase in hippocampal cofilin-2 levels. Furthermore, both memantine and donepezil significantly reduced hippocampal cofilin-2 levels in APP/PS1 mice. Collectively, these results suggest that cofilin-2 is a promising drug target and may be valuable for identifying potential therapeutic agents. Compounds that inhibit cofilin-2 could potentially alleviate learning and memory deficits in AD.

In recent decades, numerous studies have consistently demonstrated the critical role of oxidative stress and inflammatory responses in the pathogenesis of AD (Kosenko et al., 2014). Peroxiredoxin-6, a major antioxidant enzyme in human neural tissue, has been shown to play a significant role in neuroprotection (Power et al., 2008). Recent evidence indicates that the peroxidase activity of peroxiredoxin-6 protects PC12 cells from Aβ_25-35_-induced neurotoxicity (Kim et al., 2013), and that the upregulation of peroxiredoxin-6 can modulate astroglial and microglial activation around Aβ plaques, thereby mediating the protective function of Aβ proteostasis (Pankiewicz et al., 2020). In our current study, we observed a significant elevation in the peroxiredoxin-6 protein level in the cortex and hippocampus of APP/PS1 mice compared to WT mice. Consistent with these findings, increased levels of peroxiredoxin-6 have been reported in the brain tissues of amyloid transgenic mouse models (Guerreiro et al., 2008) and AD patients (Sultana et al., 2007; Power et al., 2008). This increased expression of peroxiredoxin-6 may represent a compensatory and protective response to the oxidative stress induced by Aβ accumulation. Our results indicate that peroxiredoxin-6 was upregulated following dl-PHPB treatment, suggesting that dl-PHPB may exert its therapeutic effects in APP/PS1 mice through the antioxidant properties of peroxiredoxin-6.

The major pathologic characteristics of AD encompass the accumulation of intracellular neurofibrillary tangles, the deposition of extracellular senile plaques, and neuronal loss. Pin 1 levels are significantly diminished in the cortex and hippocampus of APP/PS1 mice, a finding consistent with prior studies in AD brains (Sultana et al., 2006; Sultana et al., 2007). Pin 1 prevents tau hyperphosphorylation by inhibiting GSK3β activity (Ma et al., 2012) and promotes tau dephosphorylation through interaction with protein phosphatase 2A (PP2A) (Zhou et al., 2000), thereby restoring its function in microtubule binding. Recent studies have demonstrated that Pin 1 facilitates the non-amyloidogenic processing of APP by accelerating its cis and trans conformational changes, thus protecting against Aβ toxicity (Pastorino et al., 2012). Moreover, Pin 1 interacts with several proteins involved in cell cycle regulation (Zhou et al., 2000; Chen et al., 2012). Consequently, a decline in Pin 1 protein levels may disrupt the balanced phosphorylation state of tau proteins, leading to Aβ deposition and neuronal loss in AD (Wang et al., 2020). In our study, the reduction in Pin 1 levels was reversed by long-term treatment with dl-PHPB, indicating that dl-PHPB may exert therapeutic effects by modulating tau phosphorylation, Aβ production, and neuronal apoptosis.

Lysosomal activation is prominently observed in the pathology of AD (Bendiske and Bahr, 2003). Cathepsin B, a lysosomal cysteine protease, plays a crucial role in degrading proteins that enter the lysosomal system and has been implicated in Aβ processing and AD pathogenesis (Haque et al., 2008; Hook et al., 2020). Specifically, cathepsin B cleaves APP at the β-secretase cleavage site and enhances β-secretase-mediated cleavage of APP, contributing to the production of neurotoxic Aβ peptides (Klein et al., 2009; Schechter and Ziv, 2011). Moreover, genetic knockout (Hook et al., 2009; Kindy et al., 2012) and chemical inhibition (Hook et al., 2008; Cho et al., 2013) studies in cellular and animal models of AD have demonstrated that inhibiting cathepsin B reduces Aβ burden and improves cognitive function. Our current proteomic analysis indicates that dl-PHPB significantly decreases the expression of cathepsin B in the cortex and hippocampus of APP/PS1 mice. Previous research from our group has shown that dl-PHPB treatment markedly reduces Aβ plaque deposition in the brain tissues of APP/PS1 mice (Peng et al., 2014). Therefore, we propose that dl-PHPB may exert neuroprotective effects against Aβ plaques, at least in part, by downregulating cathepsin B levels.

Memantine, an NMDA receptor antagonist, and donepezil, an acetylcholinesterase inhibitor, are currently utilized to treat AD. As a result, they were selected as positive controls to evaluate the therapeutic efficacy of dl-PHPB in the step-down passive avoidance test. In this study, treatment with memantine, donepezil, and dl-PHPB significantly alleviated cognitive dysfunction in APP/PS1 mice. Furthermore, Western blot analysis indicated that most AD-related proteins examined in this study (PDHE1α, DRP-2, peroxiredoxin-6, Pin 1, and cathepsin B) were not modulated by memantine or donepezil, likely due to their nature as single-target drugs. However, the altered expression levels of cofilin-2 and VDAC1 in the brains of APP/PS1 mice were restored to near-normal levels by treatment with dl-PHPB, memantine, and donepezil. This suggests that cofilin-2 and VDAC1 may be involved in common AD-related signaling pathways regulated by these three drugs. Therefore, they are more likely to serve as potential biomarkers for assessing therapeutic efficacy during drug development.

In conclusion, proteomics is recognized as a valuable tool for analyzing protein alterations in complex diseases such as AD. By employing a 2DE-based proteomic approach, we have successfully identified novel candidate proteins involved in AD development and delineated potential intervention targets of dl-PHPB in the cortex and hippocampus of an AD mouse model. Notably, dl-PHPB was found to mitigate the changes in many proteins that were differentially expressed in APP/PS1 mice, suggesting that dl-PHPB may restore these alterations towards normal levels. Due to its capacity to target multiple pathways, dl-PHPB can enhance mitochondrial function, reduce oxidative stress, increase synaptic plasticity, maintain normal neuronal connections, and inhibit tau phosphorylation, Aβ production, and cell apoptosis. Treatment with dl-PHPB, memantine, and donepezil, respectively, could reverse the expression of VDAC1 and cofilin 2 to near-normal levels, indicating that these two proteins may serve as candidate biomarkers for assessing therapeutic efficacy in AD. While this study advances our understanding of VDAC1 and cofilin-2 in AD, several limitations warrant consideration. First, our findings are derived from preclinical models; thus, validation in human-derived samples or longitudinal clinical cohorts is essential to confirm translational relevance. Further studies should: (1) define the precise molecular interplay between VDAC1-mediated mitochondrial permeability and cofilin-2-regulated actin dynamics in AD pathogenesis, (2) validate their diagnostic/therapeutic utility as therapeutic targets or biomarkers in clinical cohorts, and (3) assess the therapeutic potential of pharmacological modulation strategies targeting the VDAC1/cofilin-2 pathway in AD treatment.

Our findings suggest dl-PHPB’s therapeutic potential in enhancing cognitive function in APP/PS1 mice, but several study limitations exist. First, the mechanistic insights derived from proteomic analyses remain correlative, and causality requires further validation using targeted experimental approaches such as pathway-specific inhibitors or genetic models. Second, the current study focused on a single dose; future work should explore dose-dependent effects. Third, although our behavioral tests showed significant improvements, additional neuropathological markers (e.g., synaptic plasticity, Aβ plaque load) could strengthen the conclusions. Finally, the generalizability of our findings may be limited to the specific APP/PS1 mice used here, and validation in aged or other transgenic models with advanced neurodegeneration would be valuable.

## Data Availability

The original contributions presented in the study are included in the article/supplementary material, further inquiries can be directed to the corresponding author.
